# Comprehensive analysis of hypoxia-related genes for prognosis value, immune status, and therapy in osteosarcoma patients

**DOI:** 10.3389/fphar.2022.1088732

**Published:** 2023-01-06

**Authors:** Tao Han, Zhouwei Wu, Zhe Zhang, Jinghao Liang, Chuanpeng Xia, Hede Yan

**Affiliations:** ^1^ Department of Orthopedics, The Second Affiliated Hospital and Yuying Children’s Hospital of Wenzhou Medical University, Wenzhou, China; ^2^ The Second Clinical Medical College of Wenzhou Medical University, Wenzhou, China; ^3^ Key Laboratory of Orthopedics of Zhejiang Province, Wenzhou, China

**Keywords:** hypoxia, subtype, prognostic model, osteosarcoma, immune microenvironment, chemotherapy, KCNJ3

## Abstract

Osteosarcoma is a common malignant bone tumor in children and adolescents. The overall survival of osteosarcoma patients is remarkably poor. Herein, we sought to establish a reliable risk prognostic model to predict the prognosis of osteosarcoma patients. Patients ’ RNA expression and corresponding clinical data were downloaded from the Therapeutically Applicable Research to Generate Effective Treatments (TARGET) and Gene Expression Omnibus databases. A consensus clustering was conducted to uncover novel molecular subgroups based on 200 hypoxia-linked genes. A hypoxia-risk models were established by Cox regression analysis coupled with LASSO regression. Functional enrichment analysis, including Gene Ontology annotation and KEGG pathway analysis, were conducted to determine the associated mechanisms. Moreover, we explored relationships between the risk scores and age, gender, tumor microenvironment, and drug sensitivity by correlation analysis. We identified two molecular subgroups with significantly different survival rates and developed a risk model based on 12 genes. Survival analysis indicated that the high-risk osteosarcoma patients likely have a poor prognosis. The area under the curve (AUC) value showed the validity of our risk scoring model, and the nomogram indicates the model’s reliability. High-risk patients had lower Tfh cell infiltration and a lower stromal score. We determined the abnormal expression of three prognostic genes in osteosarcoma cells. Sunitinib can promote osteosarcoma cell apoptosis with down-regulation of KCNJ3 expression. In summary, the constructed hypoxia-related risk score model can assist clinicians during clinical practice for osteosarcoma prognosis management. Immune and drug sensitivity analysis can provide essential insights into subsequent mechanisms. KCNJ3 may be a valuable prognostic marker for osteosarcoma development.

## Introduction

Osteosarcoma is widely acknowledged as the most common primary bone tumor, especially in adolescents (.8–1.1 per 100,000 per year at age 15–19 years) (Whelan et al., 2012; Valery et al., 2015). Osteosarcoma tends to occur in the metaphysis of long bones, with a high metastasis and recurrence rate. It is well-established that the lungs represent the most common metastatic site, posing a severe threat to the health of adolescents (Casali et al., 2018). Surgery, radiotherapy (RT), and multimodal chemotherapy (ChT) remain the mainstay of treatment for osteosarcoma patients (Casali et al., 2018). Despite recent advances in medicine, the prognosis of osteosarcoma is still poor. It has been reported that the aggregate event-free survival rate of patients with recurrent, unresectable osteosarcoma was only 12% at 4 months (Lagmay et al., 2016). In addition, due to genetic heterogeneity, patients with the same clinical manifestations and pathological types often have different prognoses after receiving the same treatment scheme (Wu et al., 2020). Therefore, a reliable new prognostic model is pivotal to ensuring targeted therapy and improving osteosarcoma treatment efficacy, which aligns with the concept of precision medicine.

Over the past years, experts have attached great importance to hypoxia, a feature of the tumor immune microenvironment. The rapid proliferation of cancer cells, desmoplastic fibrotic stroma, and non-functional angiogenesis lead to increased oxygen consumption and decreased oxygen supply (Gola et al., 2020; Tao et al., 2021a). Ample evidence has confirmed that tumor hypoxia has an indispensable effect on apoptosis, cell proliferation, vascularization, immune responses, metabolism, genomic instability, and metastasis (Wigerup et al., 2016). Moreover, hypoxia promotes dihydropyrimidine dehydrogenase expression in macrophages, resulting in chemotherapy resistance in colorectal cancer (Malier et al., 2021). It is generally believed that bone is prone to hypoxia. An increasing body of studies has shown that hypoxia in the tumor microenvironment is a driver for resistance to chemotherapy, tumor growth, cell survival, and metastasis in numerous solid tumors, such as osteosarcoma (Prudowsky and Yustein, 2020; Jiang et al., 2021). Studies have shown that hypoxia can induce HIF-1α, NUSAP1, and NDUFA4L2 expression and promotes the survival, epithelial-mesenchymal transition progression, migration, and invasion of osteosarcoma cells (Xu et al., 2020; Zhang et al., 2021). Hence, we should concentrate on differences in hypoxia genes to examine the biological mechanisms of osteosarcoma and reclassify tumor subtypes.

Hypoxia plays an indispensable role in tumor development and the anti-tumor process. However, the relationship between hypoxia and osteosarcoma prognosis remains further unexplored. We conducted a comprehensive analysis of hypoxia-related genes in osteosarcoma to understand the role of hypoxia in tumor genesis and development. Herein, we retrieved genes associated with hypoxia from the MSigDB database (Zhou et al., 2021). Consensus clustering analysis is a popular method for identifying new tumor molecular subtypes (Zuccato et al., 2022). Importantly, we used hypoxia gene expression as the input of consensus clustering to place a unique molecular subtype of osteosarcoma. We established a model for estimating patient prognostic risk based on this subtype. Furthermore, we analyzed the immune microenvironment and chemosensitivity of osteosarcoma. Finally, we further studied the expression and role of some predictive genes in osteosarcoma *in vitro*.

## Materials and methods

### Data collection

84 osteosarcoma samples, containing RNA-Seq data and corresponding clinical information, were downloaded from the Therapeutically Applicable Research to Generate Effective Treatments (TARGET) database (https://ocg.cancer.gov/programs/target). The clinical characteristics of the samples are shown in Supplementary Table S1. The osteosarcoma dataset GSE21257 was obtained from the GEO database (https://www.ncbi.nlm.gov/geo) as a validation cohort. The hypoxia-linked genes (shown in Supplementary Table S2) were retrieved from the hallmark gene sets in the Molecular Signature Database3 (MSigDB).

### Cluster analysis based on hypoxia-related genes

The R package “ConsensusClusterPlus” was performed to cluster the samples based on mRNA expression levels of 200 hypoxia-related genes. The lowest intergroup correlation and the highest intragroup correlation were shown when clustering variable (k) = 2. R packages “survival” and “survminer” (v3.6.1) were used to analyze the overall survival time between the two subtypes. The clinical characteristics and related genes between the two clusters were displayed in a heatmap using the R package.

### Development and validation of a hypoxia subtype-related gene prognostic model

23 differentially expressed genes (DEGs) between the hypoxia-related subtypes were identified for downstream analyses using the screening criteria |log2FC| ≥ 2 and FDR < .05. We uncovered genes related to individual survival and prognosis of osteosarcoma through Cox regression analysis in the TARGET cohort. A *p*-value of .05 was set as the threshold. LASSO (Least absolute shrinkage and selector operation) Cox regression was conducted to uncover significant prognostic genes for prognostic model using the R package “glmnet”. Ultimately, we retained the 12 genes coupled with their coefficients, and the penalty parameter (*λ*) was determined *via* the minimum criteria. The risk score formula was calculated as follows:

$$Risk\;Score=\overset{n}{\underset{i=1}{\sum }}Coef_{i}*x_{i}$$

According to the median risk score, osteosarcoma patients from the TARGET cohort were stratified into low- and high-risk groups. Based on the 12-gene signature, we performed the PCA (principal component analysis) and t-SNE (t-distributed stochastic neighbor embedding). The Kaplan-Meier analysis was conducted to compare survival possibility and overall survival time between the high- and low-risk groups. The R package “timeROC” was used to draw the time-based ROC curve. The area under the curve (AUC) was calculated to assess the sensitivity and specificity of the risk score system. Additionally, we underwent external validation for the gene signature model in the GSE21257.

### Independent prognostic analysis and a nomogram

We applied the univariate and multivariable Cox regression analysis (“survival” R package) to assess the risk score combined with clinical information (age, gender and metastatic status) in the TARGET cohort. Combined with prognostic signature, a nomogram was presented to predict 1, 3, and 5-year overall Survival (OS) of osteosarcoma patients.

### Tumor immune microenvironment and functional enrichment analysis

We employed the single sample gene set enrichment analysis (ssGSEA) in low- and high-risk groups to explore the infiltrating scores of immune cells and immune pathways. Benjamini-Hochberg (BH) correction method was used to calculate the adjusted *p*-value. We utilized the estimate algorithm to calculate the infiltration levels of immune and stromal cells. We applied Spearman correlation analysis to analyze the relationships between risk score and immune and stromal cells. Screening of DEGs between the low- and high-risk groups was done using the criteria |log2FC|≥1 and FDR < .05. The package “clusterprofiler” was used for Gene Ontology (GO) annotation and Kyoto Encyclopedia of Genes and Genomes (KEGG) pathway analysis.

### Drug sensitivity analysis

We downloaded the NCI-60 human cancer cell lines from the CellMiner database (https://discover. nci.nih.gov/cellminer) (Luna et al., 2021). Then Pearson correlation analysis was conducted to determine the correlation between prognostic genes and drug sensitivity.

### Cell lines and cultures

We purchased one human osteoblast cell line (hFOB1.19) and two human osteosarcoma cell lines (U20S and 143B) from the National Collection of Authenticated Cell Cultures (Shanghai, China). Dulbecco’s modified Eagle’s medium (DMEM, Gibco) contains 1% penicillin/streptomycin (Thermo Fisher Scientific) and 10% fetal bovine serum (FBS). We cultured hFOB1.19 cells in DMEM at 34°C with 5% CO_2_ and U20S and 143B cells at 37°C with 5% CO_2_.

### Treatment and cell viability analysis

Osteosarcoma cells were treated with a range of concentrations of sunitinib (0, 10, 20, and 30 μM). According to operational guidelines, we used the Cell Counting Kit-8 (CCK-8) assay (MedChemExpress; NJ, the United States) to evaluate cell viability. Briefly, the second-generation osteosarcoma cells were grown on 96 well plates (5 × 10^3^ cells/well) and exposed to different concentrations of sunitinib. Osteosarcoma cells were incubated in DMEM at 37°C with 5% CO_2_ for 24 h. Next, we rinsed cells with PBS and added 10 μL CCK8 to the medium for another 2 h. Finally, a microwell plate (Thermo Fisher) reader detected the absorbance at 450 nm.

### TUNEL staining

Apoptotic DNA fragmentations of osteosarcoma cells were detected with One-step TUNEL *in Situ* Apoptosis Kit (E-CK-A321, Elabscience, Wuhan, China). Osteosarcoma cells were treated with sunitinib (0, 10, 20, and 30 μM) for 24 h. Following the manufacturer’s guidelines, cells were fixed in 4% paraformaldehyde for 30 min at room temperature and then incubated with 3% H2O2 and .2% Triton X-100 for 10 min. Next, we washed with PBS three times and stained the nuclei with DAPI. Finally, we used an Olympus fluorescence microscope (Tokyo, Japan) to observe apoptotic cells (TUNEL-positive cells).

### Western blotting

We extracted total cellular protein from osteosarcoma cells using RIPA lysis buffer with 1 mM PMSF and determined their concentrations with the BCA Protein Assay Kit (Beyotime, Shanghai, China). Next, proteins were separated by PAGE and transferred to PVDF membranes. After blocking with skim milk (5%, w/v) for 2 h at room temperature, membranes were incubated with the following primary antibodies (1: 1,000) overnight at 4°C: RASGRP2 (ABclonal, A15381), KCNJ3 (Abcam, ab129182), ACTG2 (Abcam, ab231802), CASP3 (Proteintech, 19677-1-AP), Bcl-2 (Proteintech, 26593-1-AP), Bax (Proteintech, 50599-2-Ig), and GAPDH (ABclonal, AC001). Membranes were then incubated with HRP-labeled IgG secondary antibody (1:2000, Beyotime, A02080) for 2 h at 25°C. Protein bands on the membrane were visualized using the ECL Plus kit (Meilunbio). Finally, the band intensity was quantified *via* Image Lab 6.1 software (BioRad, Hercules, CA, USA).

### Statistical analysis

R software (Version:3.6.1) and GraphPad Prism (Version:7.00) were conducted to perform all statistical analyses. Pearson chi‐square test, t-test, the Mann-Whitney test, and analysis of variance (ANOVA) were used to calculate and compare the two groups. a *p*-value < .05 was statistically significant.

## Results

### Tumor classification based on the hypoxia-related genes

**FIGURE 1:**
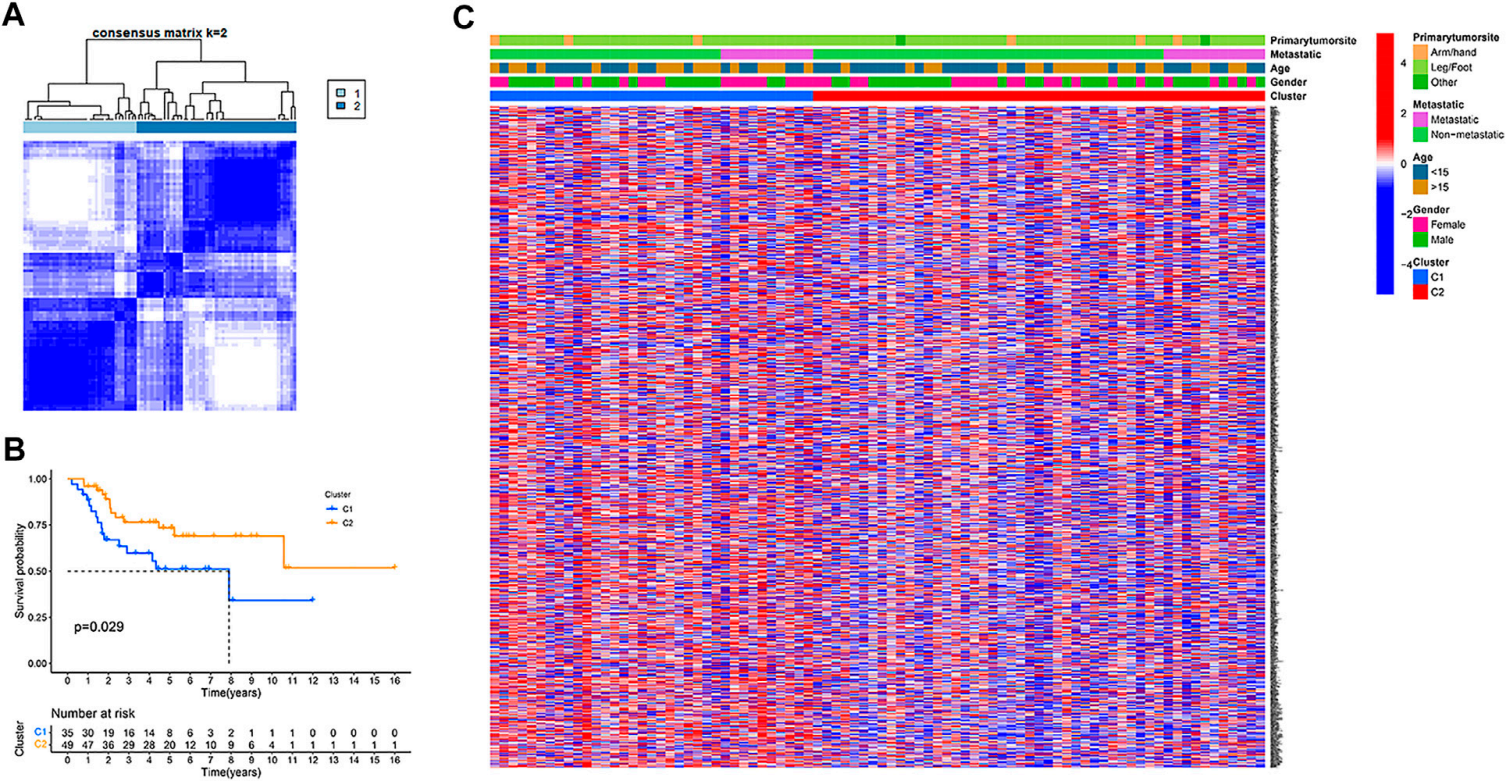
Tumour classification based on the hypoxia-related genes. **(A)** 84 osteosarcoma patients were grouped into two clusters according to the consensus clustering matrix (k = 2). **(B)** Kaplan–Meier overall survival curves for the two clusters. **(C)** A heatmap (blue: low expression level; red: high expression level) for the connections between clinicopathologic features and the clusters.

In the TARGET dataset, consensus clustering was conducted for all osteosarcoma patients to study the relationship between the expression of 200 hypoxia-related genes and osteosarcoma subtypes. After setting the clustering variable (k) value of 2, the patients could be stratified into two groups (Figure 1A). We found a significant difference in the overall survival rate between the two subtypes (*p* = .029, Figure 1B), and the cluster 2 had a significantly better overall survival than cluster 1. The relationship between the expression of these genes and the clinical characteristics, including gender, age, metastasis status, and primary tumor site, is displayed in a heatmap. However, no significant differences in clinical characteristics were observed between the two clusters (Figure 1C).

### Construction of a prognostic gene model in the TARGET cohort

**FIGURE 2:**
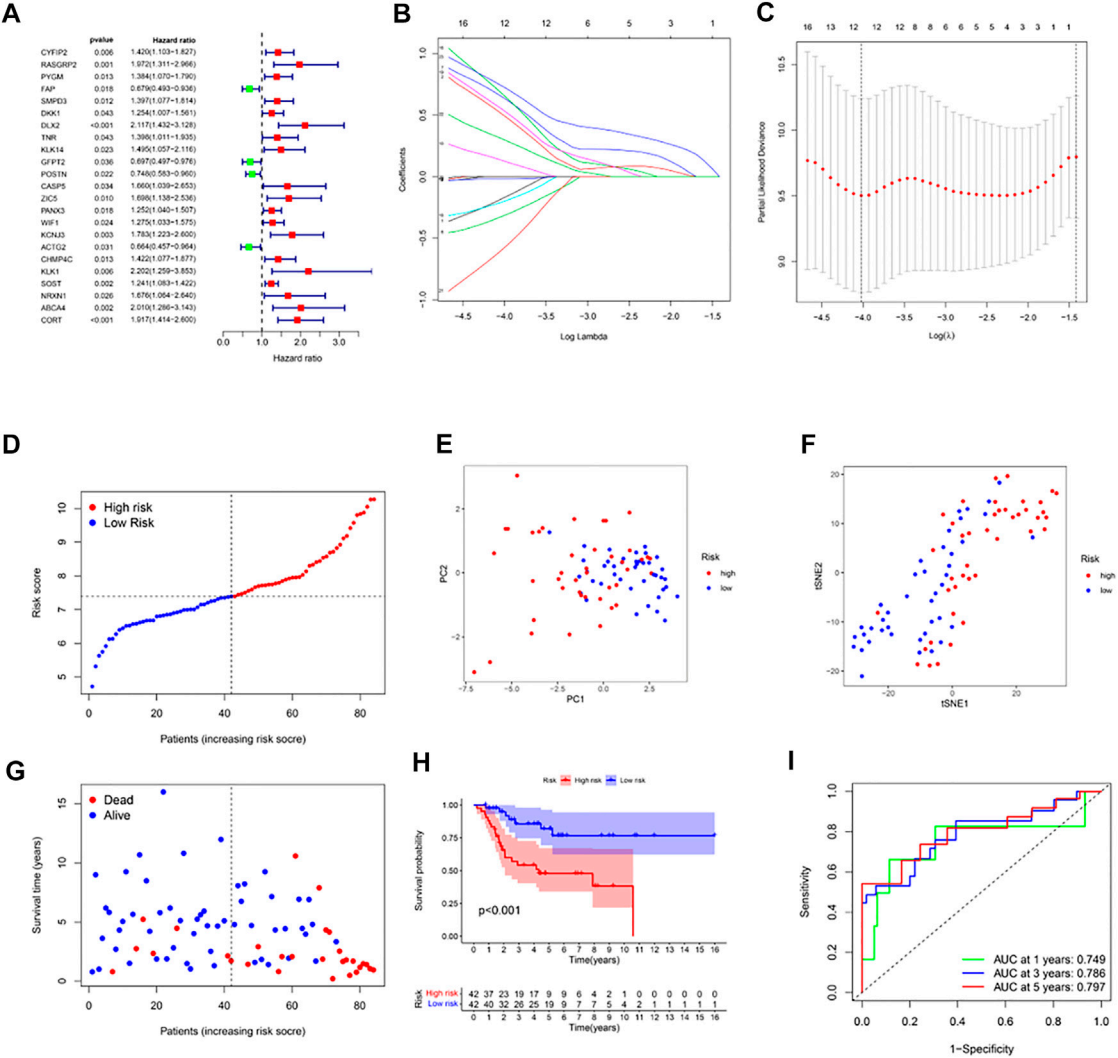
Construction of risk signature in the TARGET cohort. **(A)** Univariate cox regression analysis of overall survival for each hypoxia-related gene, and 23 genes with *p* < .05. **(B)** LASSO regression of the 12 overall survival-related genes. **(C)** Cross-validation for tuning the parameter selection in the LASSO regression. **(D)** Distribution of patients based on the risk score. **(E)** PCA plot for osteosarcoma based on the risk score. **(F)** The t-SNE analysis based on the risk score. **(G)** The survival status for each patient (left side of the dotted line: low-risk population; right side of the dotted line: high-risk population). **(H)** Kaplan–Meier curves for the overall survival of patients between the high- and low-risk groups. **(I)** ROC curves demonstrated the predictive efficiency of the risk score.

DEGs were identified between the two clusters using the screening criteria |log2FC| ≥ 2 and FDR < .05. Univariate Cox regression was used for the initial determination of survival-linked genes. 23 genes that met the criteria of *p* < .05 were retained for subsequent analyses. Of these, 4 genes (FAP, GFPT2, POSTN, ACTG2) were protective genes with an HR < 1, while the remaining 19 genes were associated with increased risk (HRs > 1) (Figure 2A). LASSO Cox regression was used to create a 12-gene signature based on the optimum *λ* value (Figures 2B, C). Computation of the risk score was done using the formula: risk score= (−.175* CYFIP2 exp.) + (−.503* RASGRP2 exp.) + (−.320* DKK1exp.) + (−.645* DLX2 exp.) + (−.130* GFPT2exp.) + (−.670* KCNJ3 exp.) + (−.019* ACTG2exp.) + (−.204* CHMP4C exp.) + (−.544* KLK1 exp.) + (−.588* NRXN1 exp.) + (−.305* ABCA4 exp.) + (−.734* CORT exp.). According to the median risk score, the 84 osteosarcoma patients were stratified into high-and low-risk subgroups (Figure 2D). PCA and the t-SNE analysis exhibited that patients with high or low risks were well separated into two classes (Figures 2E, F). In contrast with the high-risk group, the low-risk group experienced fewer deaths and exhibited longer survival (Figure 2G, left side of dotted line). The Kaplan-Meier curve showed that overall survival time and possibility were significantly lower in the high-risk group (*p* < .001, Figure 2H). Then ROC analysis was applied to determine the sensitivity and specificity of the prognostic model. We found that the value of the area under the ROC curve (AUC) was .749 for 1-year, .786 for 3-year, and .797 for 5-year survival prediction (Figure 2I).

### External validation of the risk score

**FIGURE 3:**
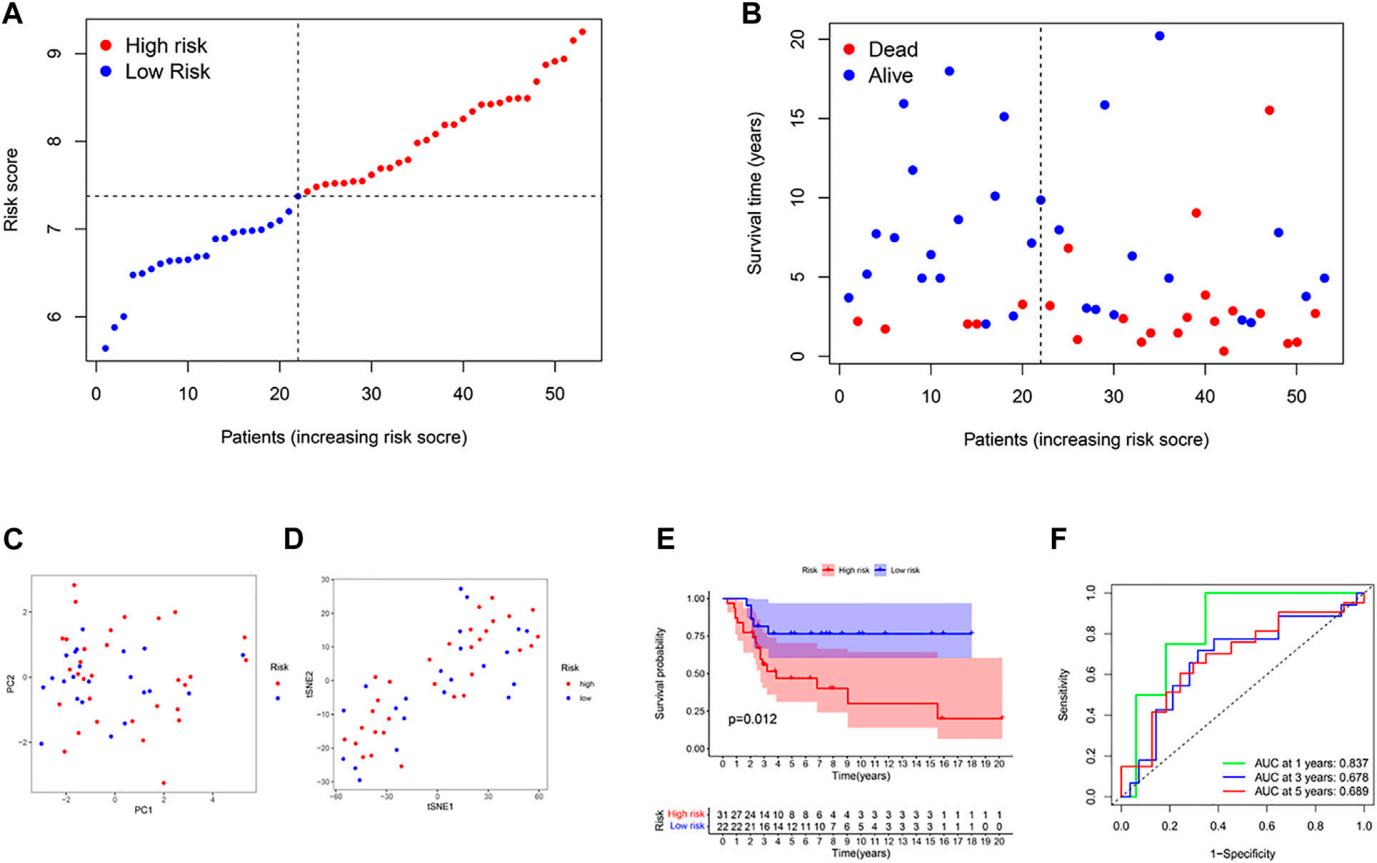
Validation of the risk model in the GEO cohort. **(A)** Distribution of patients in the GEO cohort based on the median risk score of the TARGET cohort. **(B)** The survival status for each patient (left side of the dotted line: low-risk population; right side of the dotted line: high-risk population). **(C)** The PCA plot for osteosarcoma. **(D)** The t-SNE analysis based on the risk score. **(E)** Kaplan–Meier curves for comparison of the overall survival between low- and high-risk groups. **(F)** Time-dependent ROC curves for osteosarcoma.

53 osteosarcoma patients from a GEO cohort (GSE21257) were extracted as the external validation set. The gene expression data were normalized with the R function “Scale”. Based on the median risk score of the TARGET model, 28 patients were regarded as the high-risk group, while the other 25 were at low risk (Figure 3A). High-risk patients had higher mortality and shorter overall survival time (Figure 3B on the right of the dotted line). The PCA plot and the t-SNE results showed that the two groups were separated (Figures 3C, D). Besides, there were significant differences in survival time between the high-OS and low-OS risk groups (*p* = .012, Figure 3E). ROC curve analysis of the GEO dataset showed that our model had an excellent prediction power, with AUC values for survival at 1, 2, and 3 years of .837, .678, and .689, respectively (Figure 3F).

### Independent prognostic value of the risk model

**FIGURE 4:**
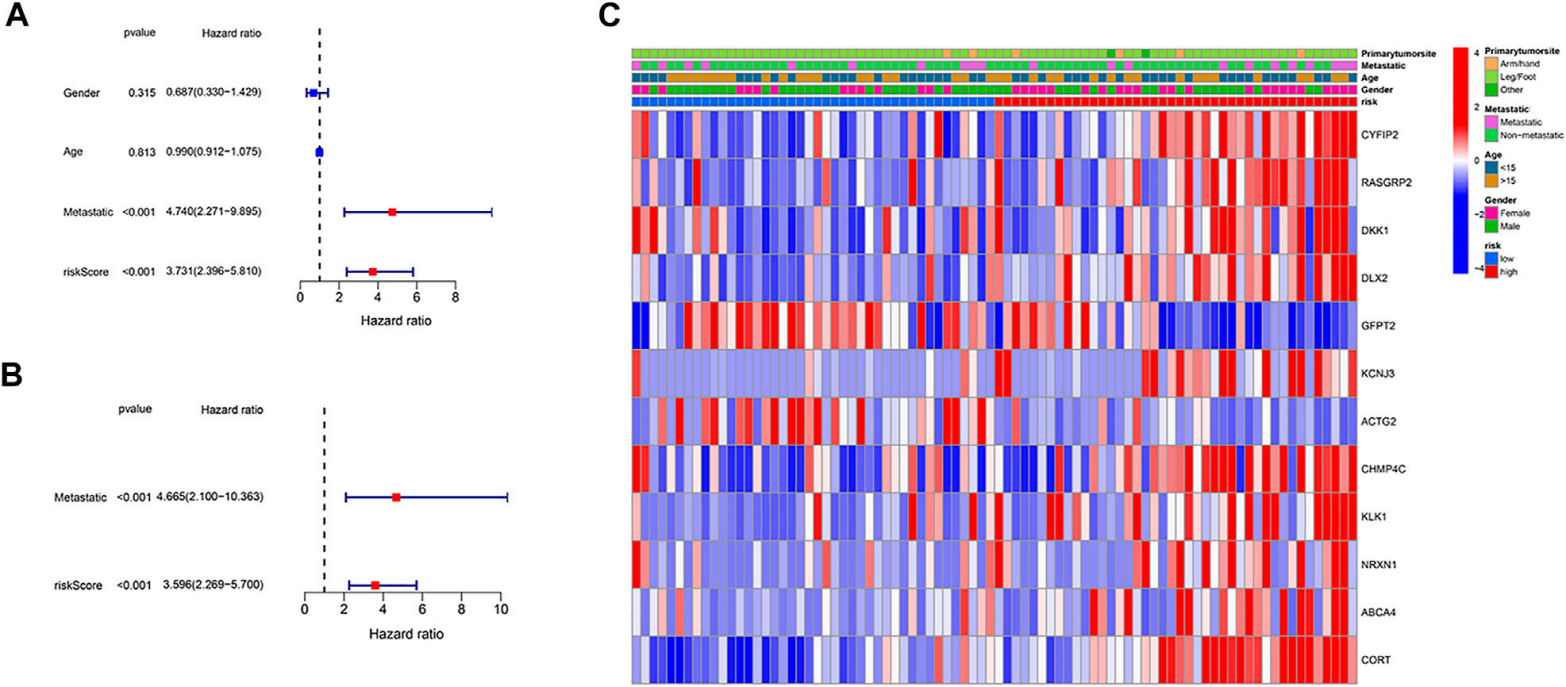
Independence detection of the constructed risk prediction model. **(A)** Univariate analysis for the TARGET cohort. **(B)** Multivariate analysis for the TARGET cohort. **(C)** A heatmap (blue: low expression; red: high expression) for the connections between clinicopathologic features and the risk groups.

We employed univariate and multivariable Cox regression to assess independent prognostic factors of the gene-based risk score and clinical characteristics. The univariate Cox regression data illustrated that the risk score and metastatic status were independent predictors of poor survival in the TARGET cohort (*p* < .001, Figure 4A). After correcting for confounding factors, the results of multivariate cox regression indicated that risk score (HR = 4.665, *p* < .001) and metastatic status (HR = 3.596, *p* < .001) were still independent predictors of the prognosis of osteosarcoma patients (Figure 4B). Moreover, we provided a heatmap to show gene expression and clinical characteristics between low- and high-risk groups (Figure 4C). Two genes (GFPT2 and ACTG2) were downregulated, while the remaining genes were upregulated in the high-risk subgroup.

### Nomogram

**FIGURE 5:**
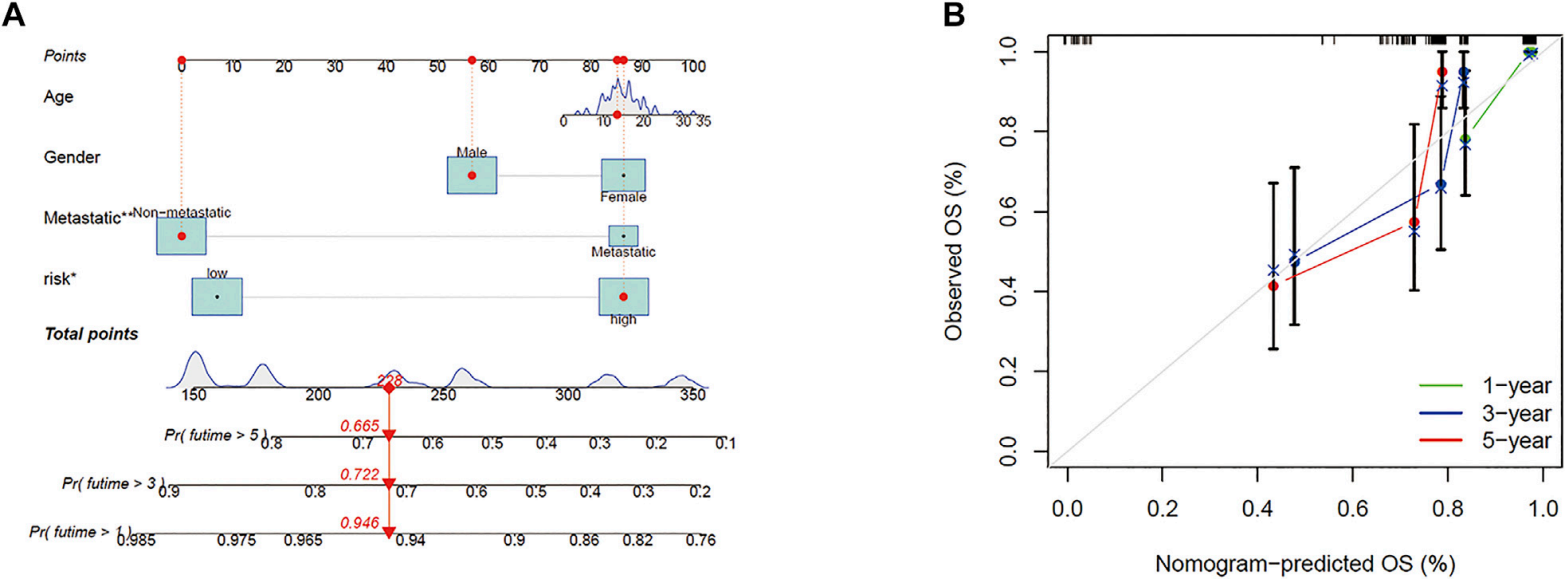
Construction of the predictive model. **(A)** A prognostic model to predict overall survival in the TARGET cohort. **(B)** Calibration curves of the OS nomogram model in the TARGET set.

According to the prognostic model and clinical factors (age, gender, and metastatic status), we developed a risk estimation nomogram in the TARGET cohort (Figure 5A). 1-, 3-, and 5-year calibration curves showed that the nomogram consistently predicted the survival rate (Figure 5B).

### Immune status and tumor microenvironment

**FIGURE 6:**
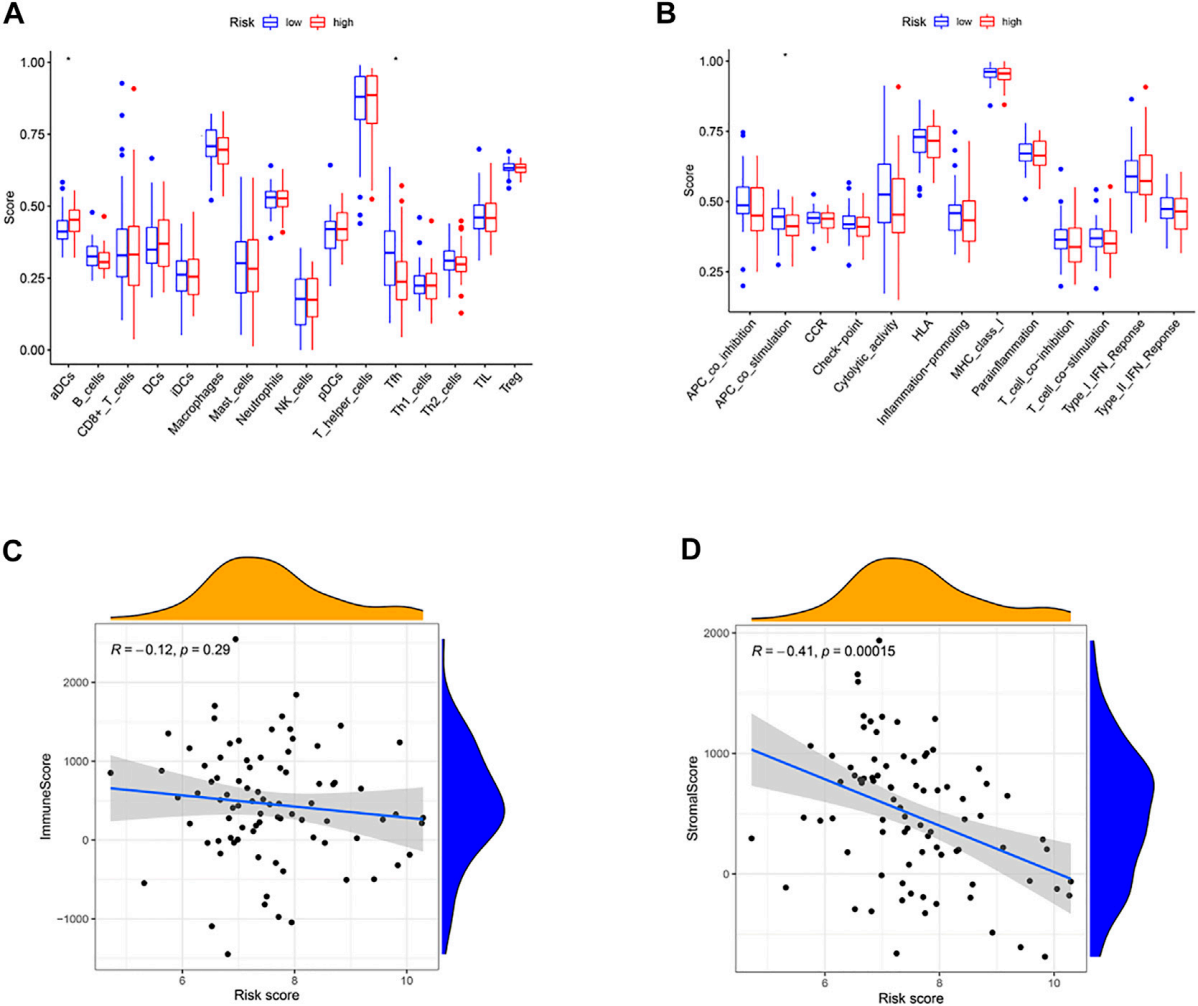
Immune status between different risk groups and the association between risk score and tumor microenvironment. **(A)** Comparison of the enrichment scores of 16 types of immune cells between low- (blue box) and high-risk (red box) group in the TARGET cohort. **(B)** Comparison of the enrichment scores of 13 types of immune functions between low- (blue box) and high-risk (red box) group in the TARGET cohort. **(C)** The relationship between risk score and immune score. **(D)** The relationship between risk score and stromal score. (*p < .05).

We calculated these two clusters’ enrichment scores of diverse immune cell sub-populations by ssGSEA. Significant differences in aDCs cells and Tfh cells were found between the high and low-risk groups (Figure 6A). Meanwhile, we indicated that the immune score of APC_co_stimulation was decreased in high-risk group, compared to the low-risk group (Figure 6B, *p* < .05). Moreover, correlation analysis illustrated that the risk score was positively correlated to stromal score (*p* < .001, Figure 6D), while no significant relationship was observed between the immune score and risk score (*p* > .05, Figure 6C).

### Functional analyses of the risk model

**FIGURE 7:**
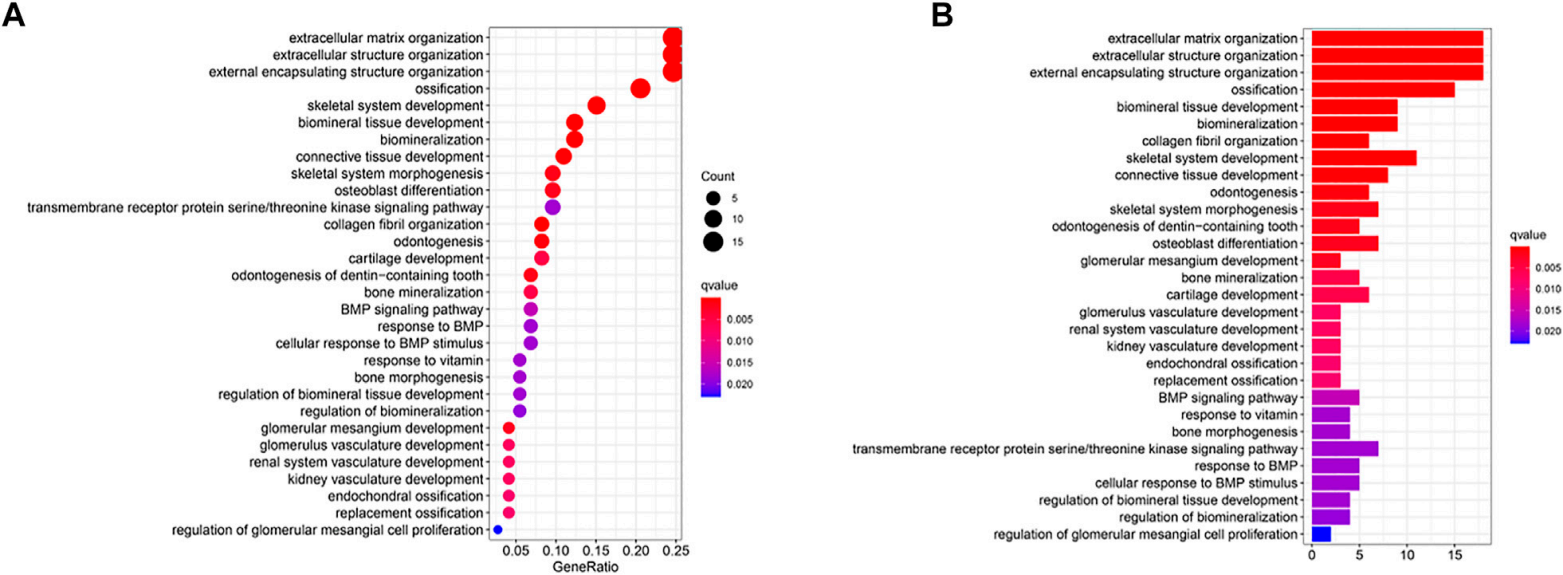
Functional analysis based on the DEGs between the two-risk groups in the TARGET cohort. **(A)** Bubble graph for GO enrichment (the bigger bubble means the more genes enriched, and the increasing depth of red means the differences were more obvious; q-value: the adjusted *p*-value). **(B)** Barplot graph for KEGG pathways (the longer bar means the more genes enriched, and the increasing depth of red means the differences were more obvious).

To further assess differences in gene functions and cascades between the sub-clusters classified *via* the risk model, the R package “limma” was used to screen DEGs with an FDR < .05 along with |log2FC |≥1. We identified 73 DEGs between low-OS and high-OS risk groups in the Target cohort. Of these, 50 genes were upregulated, and 23 genes were downregulated in the high-risk group (Supplementary Table S3). These DEGs underwent GO and KEGG analyses. We found that biological processes linked to hypoxia (i.e., organization of extracellular matrix, organization of extracellular structure, and organization of external encapsulating structure) were significantly enriched. (Figures 7A, B).

### Sensitivity to chemotherapy

**FIGURE 8:**
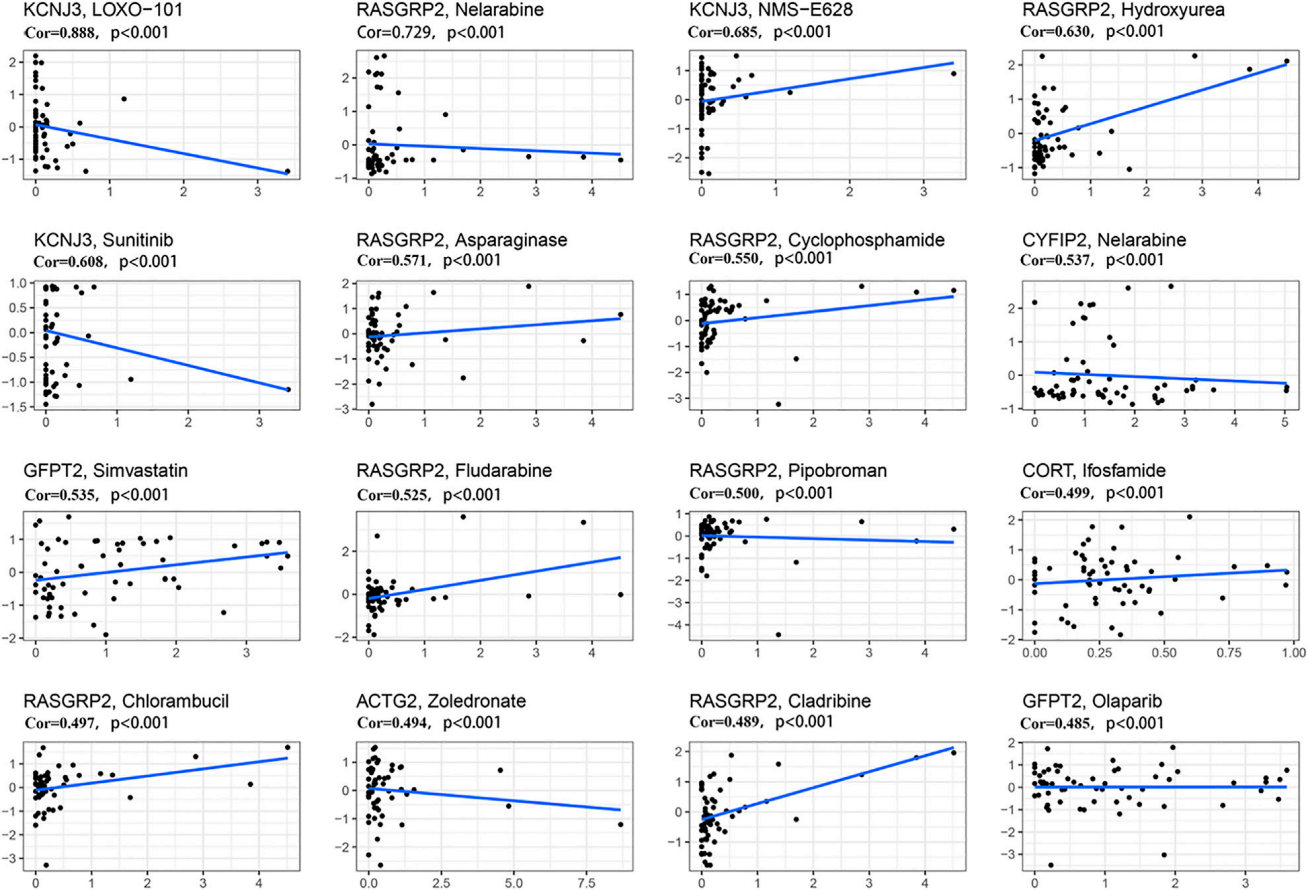
Scatter plot of relationship between prognostic gene expression and drug sensitivity. The top 16 correlation analyses are shown based on the *p*-value. The horizontal axis represents the gene expression; The vertical axis represents changes in gene expression after administration.

We further investigated the sensitivity of 12 predictive hypoxia-related genes to chemotherapeutic drugs. At the same time, we downloaded data from the NCI-60 panel of human cancer cell lines. Results for the top 16 correlation analysis were shown based on the *p*-value (Figure 8). KCNJ3 is sensitive to multiple chemotherapeutic agents, including LOXO-101, NMS-E628, and sunitinib (all *p* < .001). Results showed that RASGRP2 is sensitive to nelarabine, hydroxyurea, asparaginase, cyclophosphamide, fludarabine, pipobroman, chlorambucil, and cladribine (all *p* < .001). Besides, ACTG2 is susceptible to zoledronate (*p* < .001). Please see Supplementary Table S4 for details.

### Detection of three predictive hypoxia-related markers in osteosarcoma

**FIGURE 9:**
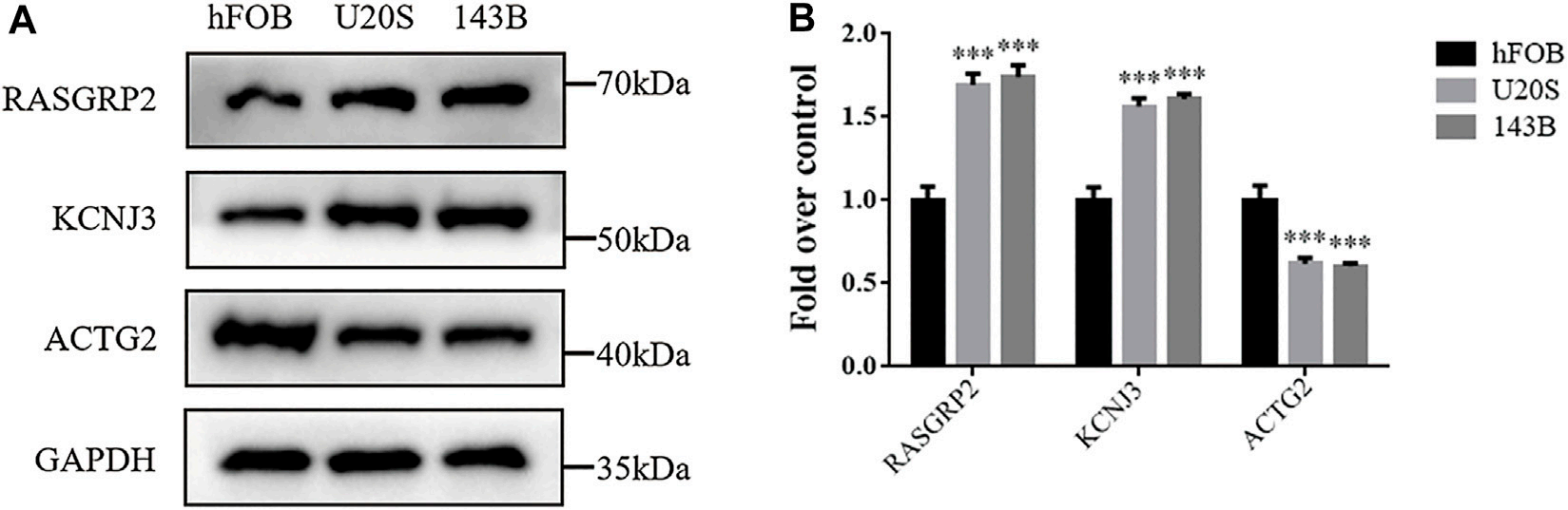
The expression levels of three hypoxia-related genes between osteosarcoma cell lines and osteoblasts. **(A)** Western blotting of the expressions of RASGRP2, KCNJ3, and ACTG2 in hFOB, U20S, and 143B groups. GAPDH serves as an internal standard. The gels have been run under the same experimental conditions. **(B)** A histogram of the OD values of RASGRP2, KCNJ3, and ACTG2 in each group (*n* = 3 per group). The obtained data are represented as mean ± SE. Significance: ***p-value < .001, vs. hFOB group.

We investigate the expression levels of three hypoxia-related genes in osteosarcoma cells using western blotting analysis. Results showed higher expression levels of RASGRP2 and KCNJ3 in two osteosarcoma cell groups (U20S and 143B) compared to the osteoblast cell group (hFOB). In contrast, ACTG2 was down-regulated in osteosarcoma groups (Figures 9A, B). Such results validated the abnormal expression of three prognostic genes in osteosarcoma.

### Sunitinib down-regulated KCNJ3 expression and promoted apoptosis in osteosarcoma cells

**FIGURE 10:**
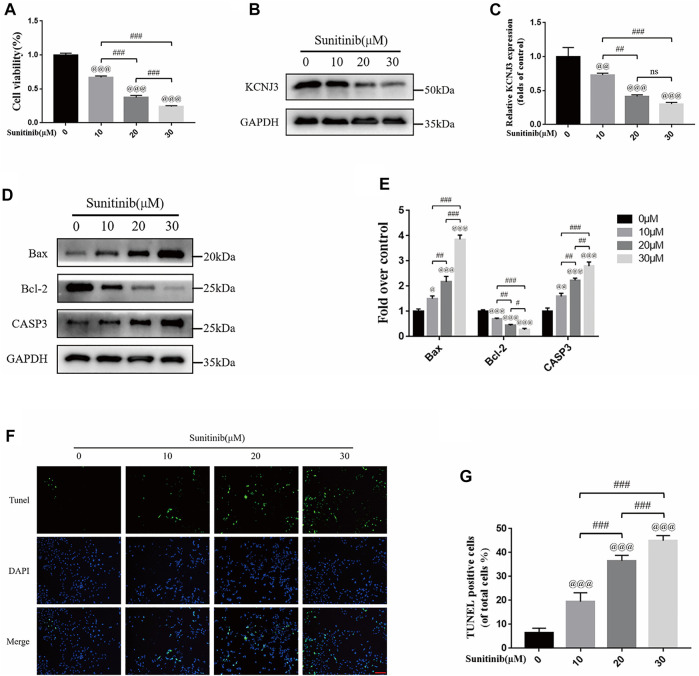
Sunitinib can decrease KCNJ3 expression and enhance apoptosis in U20S osteosarcoma cells. **(A)** Evaluation of U20S osteosarcoma cell viability using CCK-8 assay after exposure to various concentrations of sunitinib for 24 h. **(B,C)** The expression level of KCNJ3 protein of osteosarcoma cells in control and sunitinib treatment groups. **(D,E)** The protein expression levels of Bax, Bcl-2, and caspase3 in osteosarcoma cells in the sham (0μM) and sunitinib treatment groups. **(F,G)** TUNEL staining was used to detect osteosarcoma cell apoptosis after sunitinib treatment (bar: 50 μm; nuclei: blue; positive cells: green). All experiments were repeated in triplicates (*n* = 3). The obtained data are represented as mean ± SE. Significance: ^@^ *p*-value < .05, ^@@^ *p*-value < .01, ^@@@^ *p*-value < .001, vs. sham (0 μM) group. ^#^ *p*-value < .05, ^##^ *p*-value < .01, ^###^ *p*-value < .001.

**FIGURE 11:**
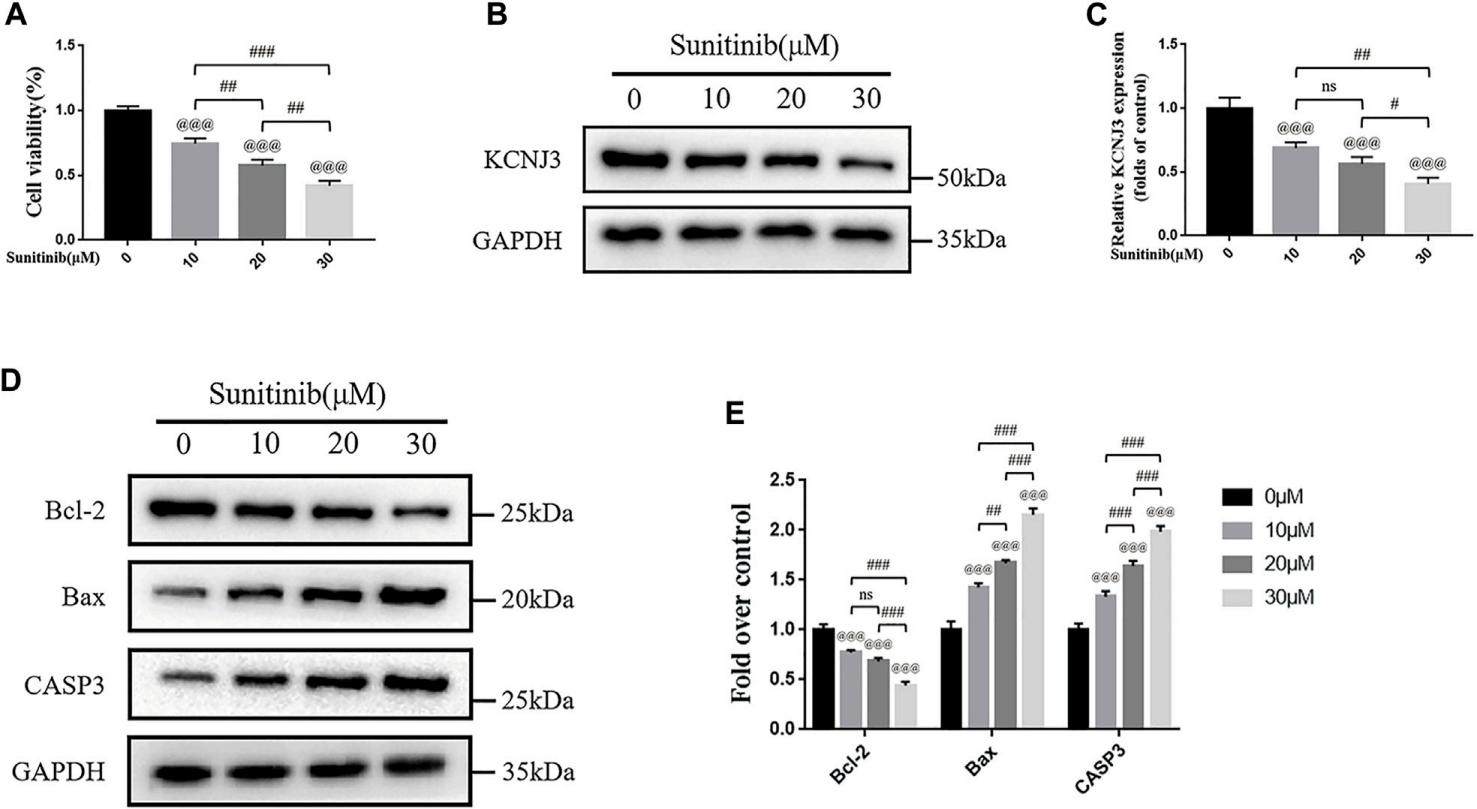
Sunitinib promotes apoptosis in 143B osteosarcoma cells with decreased expression of KCNJ3. **(A)** 143B osteosarcoma cell viability evaluation *via* CCK-8 assay. **(B,C)** The expression level of KCNJ3 protein. **(D,E)** The protein expression levels of Bcl-2, Bax, and caspase3. All experiments were repeated in triplicates (*n* = 3). The obtained data are represented as mean ± SE. Significance: ^@@@^ *p*-value < .001, vs. sham (0 μM) group. ^#^ *p*-value < .05, ^##^ *p*-value < .01, ^###^ *p*-value < .001.

To evaluate the therapeutic effect of sunitinib on osteosarcoma, we treated two types of osteosarcoma cells (U20S and 143B) with various doses of sunitinib (10, 20, or 30 μM). Sunitinib demonstrated a cytotoxic effect on osteosarcoma cells in a dose-dependent manner (Figure 10A; Figure 11A). Western blotting was used to determine the level of KCNJ3 in U20S and 143B osteosarcoma cells with various doses of sunitinib. Results indicated the expression of KCNJ3 was down-regulated after sunitinib treatment (Figures 10B, C; Figures 11B, C). Western blot results demonstrated that the expression levels of Bax and CASP3 were enhanced with the increasing sunitinib concentration, while the expression of Bcl-2 decreased (Figures 10D, E); (Figures 11D, E). Additionally, TUNEL was used to assess sunitinib treatment for osteosarcoma cells. As expected, the number of TUNEL-positive cells was upregulated with the increase of sunitinib concentration (Figures 10F, G).

## Discussion

Osteosarcoma is the most common primary tumor with early metastasis and rapid progression, accounting for the poor survival rates (Simpson et al., 2017). Despite remarkable medical advancements achieved in recent years, little progress has been made in improving osteosarcoma patients’ prognosis, considering that the current clinical approach is still based on conventional methods and is often ineffective (Ouyang et al., 2020). Precision medicine is widely acknowledged as a medical model that adjusts disease prevention and treatment methods according to the individual differences of each patient. In recent years, with the rapid development of tumor molecular biology and genomics, the understanding of tumor molecular phenotype has been improving. Accordingly, the development of targeted therapy based on specific molecular phenotypes has become the preferred treatment method for advanced cancer, accounting for the increase in the momentum of precision medicine for clinical tumor treatment (Konig et al., 2017). High throughput sequencing is the cornerstone of precision medicine. A comprehensive understanding of tumor molecular phenotype is significant for rapid and accurate diagnosis, treatment efficacy, and prognosis prediction (Heather and Chain, 2016). An increasing body of evidence shows that hypoxia is indispensable in promoting survival, epithelial-mesenchymal transition (EMT) progression, invasion of osteosarcoma cells, and drug resistance (Prudowsky and Yustein, 2020; Fu et al., 2021; Li et al., 2022a). Therefore, in this study, patients with osteosarcoma were classified based on the expression of hypoxia-related genes.

Based on these 200 hypoxia-related genes, osteosarcoma patients were divided into C1 and C2 types to determine the differentially expressed prognostic genes. To further evaluate the prognostic significance of these genes, univariate Cox regression and LASSO Cox regression analysis were applied to establish a 12-gene risk signature. The good performance of the signature was validated in an external dataset. CYFIP2 is well-recognized as a p53-driven pro-apoptotic protein with low expression levels in gastric cancer (Jackson et al., 2007; Jiao et al., 2017). Similar to the conclusions of He Y et al. (He et al., 2021), we believe that RASGRP2 is a risk factor for the prognosis of osteosarcoma patients. RASGRP2 can inhibit apoptosis by activating Rap1 to down-regulate the production of TNF-induced ROS (Takino et al., 2019). Another study demonstrated that DKK1 upregulation could accelerate the deterioration of bone microstructure related to the occurrence of femoral head necrosis and osteosarcoma (Chen et al., 2021). DLX2, a homeobox transcription factor, plays an indispensable role in tumor progression and metastasis in TGF-β-exposed cancer cells (Park et al., 2021). Overexpression of DLX2 has been documented to promote osteoblast differentiation (Zeng et al., 2020). Moreover, it has been reported that GFPT2 is involved in the developing colorectal cancer (Liu et al., 2020). Besides, the potassium channel gene KCNJ3 has been upregulated in non-small cell lung cancer, pancreatic cancer, and breast cancer. KCNJ3, a gene encoding G-protein activated inwardly rectifying K (+) channel (GIRK1), is related to lymph node metastasis and prognosis in breast cancer patients (Kammerer et al., 2016; Rezania et al., 2016). In addition, the differential expression of ACTG2 may play an essential role in the development of osteosarcoma (Lauvrak et al., 2013). Moreover, CHMP4C promotes the viability and motility of cervical cancer cells by regulating epithelial-mesenchymal transition (Lin et al., 2020). KLK1, a member of the Kalinin gene family, can increase extracellular matrix degradation and enhance tumor cells’ survival, proliferation, and invasion (Avgeris et al., 2012). Interestingly, the cell adhesion molecule neurexin-1 (NRXN1) is considered a new potential target for tumor cells (Li et al., 2022b; Feng et al., 2022). ABCA4 has been extensively studied in retinal diseases (Scortecci et al., 2021), with few studies on tumors warranting further research. We performed immunoblot *in vitro* for RASGRP2, KCNJ3, and ACTG2 genes to further verify our results’ reliability. The expression levels of RASGRP2 and KCNJ3 were upregulated in osteosarcoma cells, while the expression of ACTG2 was decreased.

Over the years, few studies have focused on immunotherapy and immune cell infiltration for osteosarcoma. Accordingly, exploring the immune cell infiltration and immune status in osteosarcoma is crucial to better understanding the molecular mechanism. By comparing the immune cell infiltration in the low-and high-risk groups, we found that the infiltration level of aDCs in the high-risk group was significantly higher. In comparison, the infiltration level of Tfh cells was considerably lower. Consistently, Tao Z et al. found that the expression of Tfh in thymoma tissues decreased while the expression of aDCs increased (Tao et al., 2021b). Indeed, the primary function of Tfh cells is to participate in information transmission during the process of B-cell differentiation and assist in B-cell activation. Tfh cells are abundant in inflammatory infiltrates of breast cancer, and the presence of Tfh cells can improve survival and reduce immunosuppression (Gu-Trantien et al., 2013). Besides, regulating Tfh cell infiltration may provide a therapeutic way for colorectal cancer treatment (Overacre-Delgoffe et al., 2021). Accordingly, we believe that high infiltration levels of Tfh cells in tumor tissues may benefit osteosarcoma patients’ prognosis. Overall, these results indicate that the tumor immune microenvironment plays an important role in tumor therapy.

We further conducted GO and KEGG pathway enrichment analysis to understand the possible biological functions of these prognostic genes. Our results illustrated that these genes were remarkably abundant in the extracellular matrix and extracellular structures, indicating a significant correlation between hypoxia-related genes and osteosarcoma prognosis. Subsequently, we evaluated gene therapy targets’ sensitivity to drugs for future treatment. Based on data analysis of sixty different cell lines, the elevated expressions of 12 prognostic genes influence sensitivity or resistance to chemotherapy drugs approved by the Food and Drug Administration. For example, with the increased KCNJ3 expression, cancer cells were sensitive to LOXO-101, NMS-E628, and sunitinib. CORT up-regulation is a useful biomarker for cancer cells sensitive to ifosfamide. At present, clinicians generally use ifosfamide in osteosarcoma chemotherapy. Therefore, these findings may provide a new perspective for precision treatment for osteosarcoma.

We found that KCNJ3 expression in tumor cells significantly decreased after administering sunitinib through drug sensitivity analysis. KCNJ3 expression was reduced in osteosarcoma cells (U20S and 143B) in a dose-dependent manner of sunitinib. Meanwhile, sunitinib significantly promoted osteosarcoma cell apoptosis. One study reported that sunitinib could reduce PD-L1 expression and remodel the immune system, thus inhibiting the migration and invasion of osteosarcoma cells (Duan et al., 2020). Another study showed that sunitinib can activate immune cells for sarcoma treatment (Ocadlikova et al., 2021). Local implantation of sunitinib and chlorin e6 can promote 143B osteosarcoma cell apoptosis (Yu et al., 2020). We believed sunitinib might provide better treatment for osteosarcoma with elevated KCNJ3 expression.

Nevertheless, there are some limitations to our study. Firstly, our risk score model was constructed and validated based on public databases. Therefore, we need a multicenter extensive sample survey to evaluate the clinical application of our model. Moreover, the mechanism of these 12 predictive genes isn’t precise in osteosarcoma. We will conduct comprehensive functional experiments and multi-omics analysis in future research.

## Conclusion

In conclusion, we uncovered a novel molecular subgroup *via* consensus clustering in the present study. Subsequently, we established a prognostic model and nomogram for osteosarcoma patients to predict the prognosis based on 12 hypoxia-related DEGs. The prognostic signature reflected the different risk groups’ immune characteristics and chemotherapeutic drug sensitivity. Our model may guide the clinical application and patients’ prognostic management. Of note, KCNJ3 may be a reliable prognostic biomarker for clinical application.

## Data Availability

The original contributions presented in the study are included in the article/Supplementary Material, further inquiries can be directed to the corresponding author.
