# Supplementary material for: Comprehensive analysis of hypoxia-related genes for prognosis value, immune status, and therapy in osteosarcoma patients
Source: Front Pharmacol. 2023 Jan 6;13:1088732. doi: 10.3389/fphar.2022.1088732 (PMC9853159; doi:10.3389/fphar.2022.1088732)
Supplement: Supplementary file 3 [file Table1.DOCX]

Table S1 The clinical characteristics of osteosarcoma patients

| Characteristics | option | Patients (n) | Rate (%) |
| --- | --- | --- | --- |
| sex | Female | 37 | 44.05 |
|  | Male | 47 | 55.95 |
| Age | ≤15 | 46 | 54.76 |
|  | >15 | 38 | 45.24 |
| Disease at diagnose | Metastatic disease | 21 | 25.00 |
|  | Non-metastatic disease | 63 | 75.00 |
| Primary tumor site | Arm/hand | 6 | 7.14 |
|  | Leg/foot | 76 | 90.48 |
|  | Other | 2 | 2.38 |
| Vital status | Dead | 27 | 32.14 |
|  | Alive | 57 | 67.86 |
